# An integrated remote sensing, petrology, and field geology analyses for Neoproterozoic basement rocks in some parts of the southern Egyptian-Nubian Shield

**DOI:** 10.1038/s41598-024-62093-0

**Published:** 2024-06-26

**Authors:** Hatem M. El-Desoky, Imane Bachri, Ahmed M. El Mezayen, Ahmed M. Abdel-Rahman, Hamada El-Awny, Arafa A. El-Gammal, Fahad Alshehri, Sattam Almadani

**Affiliations:** 1 Geology Department, Faculty of Science, Al-Azhar University, PO Box 11884, Nasr City, Cairo Egypt; 2 Abdullah Alrushaid Chair for Earth Science Remote Sensing Research, Geology and Geophysics Department, King Saud University, 11451 Riyadh, Saudi Arabia; 3 Laboratory of Applied Geology, Geomatics and Environment, Faculty of Sciences Ben M’sik, Casablanca, Morocco; 4 Egyptian Mineral Resources Authority, Cairo, Egypt

**Keywords:** Neoproterozoic basement, Lithological mapping, Remote sensing, Landsat-9, PCA, Band ratio, CNN, Egypt, Petrology, Geology, Precambrian geology

## Abstract

The main objective of this study was to use deep learning, and convolutional neural networks (CNN), integrated with field geology to identify distinct lithological units. The Samadia-Tunduba region of the South Eastern Desert of Egypt was mapped geologically for the first time thanks to the use of processed developed CNN algorithms using Landsat 9 OLI-2, which were further enhanced by geological fieldwork, spectral measurements of field samples, and petrographic examination. According to previously published papers, a significant difference was observed in the distribution of rocks and their boundaries, as well as the previously published geological maps that were not accurately compatible with the nature of the area. The many lithologic units in the region are refined using principal component analysis, color ratio composites, and false-color composites. These techniques demonstrated the ability to distinguish between various igneous and metamorphic rock types, especially metavolcanics, metasediments, granodiorite, and biotite monzogranite. The Key structural trends, lithological units, and wadis affecting the area under study are improved by the principal component analysis approach (PC 3, 2, 1), (PC 2, 3, 4), (PC 4, 3, 2), (PC 5, 4, 3), and (PC 6, 5, 4) in RGB, respectively. The best band ratios recorded in the area are recorded the good discrimination (6/5, 4/3, and 2/1), (4/2, 6/7, and 5/6), and (3/2, 5/6, and 4/6) for RGB. The classification map achieved an overall accuracy of 95.27%, and these results from Landsat-9 data were validated by field geology and petrographical studies. The results of this survey can make a significant difference to detailed geological studies. A detailed map of the new district has been prepared through a combination of deep learning and fieldwork.

## Introduction

Geological mapping is of crucial importance in geological research, constituting an essential pillar for guiding mineral exploration activities^1–13^. Remote sensing is considered to be the most effective and useful technique for identifying lithological units. Satellite images contain information on the target's electromagnetic waves, and have the advantage of being multiscale, multitemporal and of high spatial resolution, particularly in areas that are difficult to access, such as highly weathered and tectonically complex arid regions^14–16^. Various approaches have been developed to identify lithological units for geological mapping. These encompass a range of approaches, from statistical techniques such as principal component analysis, to spectral analysis methods such as band ratios and spectral indices^1–6^. In addition, machine learning algorithms, both supervised and unsupervised, such as random forests (RF), support vector machines (SVM) and K-Means, have emerged as powerful tools. The integration of machine learning approaches with data from multiple sources is proving to be an efficient and cost-effective method for geological mapping, taking into account the complexity, morphological, and spectral characteristics of different geological regions, enabling improvements in the accuracy and robustness of the geological maps generated. However, the aforementioned machine learning approaches make decisions for each pixel during map classification. This could lead to a lack of consideration of the spatial characteristics of the surrounding data, potentially giving rise to a "salt and pepper" phenomenon in the classification results, thus introducing inconsistency into geological mapping.

Deep learning algorithms can overcome the limitations of these methods. Deep learning, embodied in neural network models with multiple hidden layers, stands out for its ability to increase the accuracy of classification and prediction tasks, by automatically extracting deep, abstract features from the data. Convolutional neural networks (CNNs), prominent among deep learning algorithms, manage to simulate the structure and function of neurons in the human brain. These models are particularly well known for their ability to process complex data, notably in the field of image classification^17^. Their growing popularity is due to their superior performance compared with traditional machine learning (ML) algorithms, offering more accurate and consistent results^18^.

The Egyptian Nubian Shield (ENS) of the Late Neoproterozoic has garnered significant interest ever since the Egyptian Geological Survey and Mining Authority (EGSMA) was founded in 1896 and carried out the initial survey. Rapid advancements in analytical methods and field-focused research over the past three decades have allowed for the interpretation of the ENS as a crucial component of the juvenile Arabian-Nubian Shield (ANS), a section of the East African Orogeny (EAO). The Egyptian Nubian Shield (ENS), represented by the Eastern Desert (ED) and Sinai, which is a contiguous part of the Arabian–Nubian Shield (ANS), a northern continuation of the East African Orogen (EAO). The accumulation of island arc tectonic terranes sutured along megashear zones embellished with ophiolites gave rise to the ANS. Transcurrent plate motion during the late orogenic development produced a complex network of post-accretional strike-slip and extensional shear zones. One of these tectonic terranes, the Eastern Desert (ED), showed a clear window into the Neoproterozoic ANS's magmatic, tectonic, and metamorphic history^19–22^.

Ophiolitic nappes, arc-related volcano-sedimentary layers, and post-amalgamation molasses deposits dominated the Late Neoproterozoic belt of the ED. These features were all associated with the emplacement of different magmatic suites. The infrastructural-supra structural orogenic model served as a solid foundation for the rock classification and tectonic history of the Egyptian basement in ED^22–24^. The volcaniclastic-metasediments, arc-related metavolcanics, and ophiolitic nappes made up the supra structural rocks (also known as "Tier-2" according to^25^).

**Figure 1:**
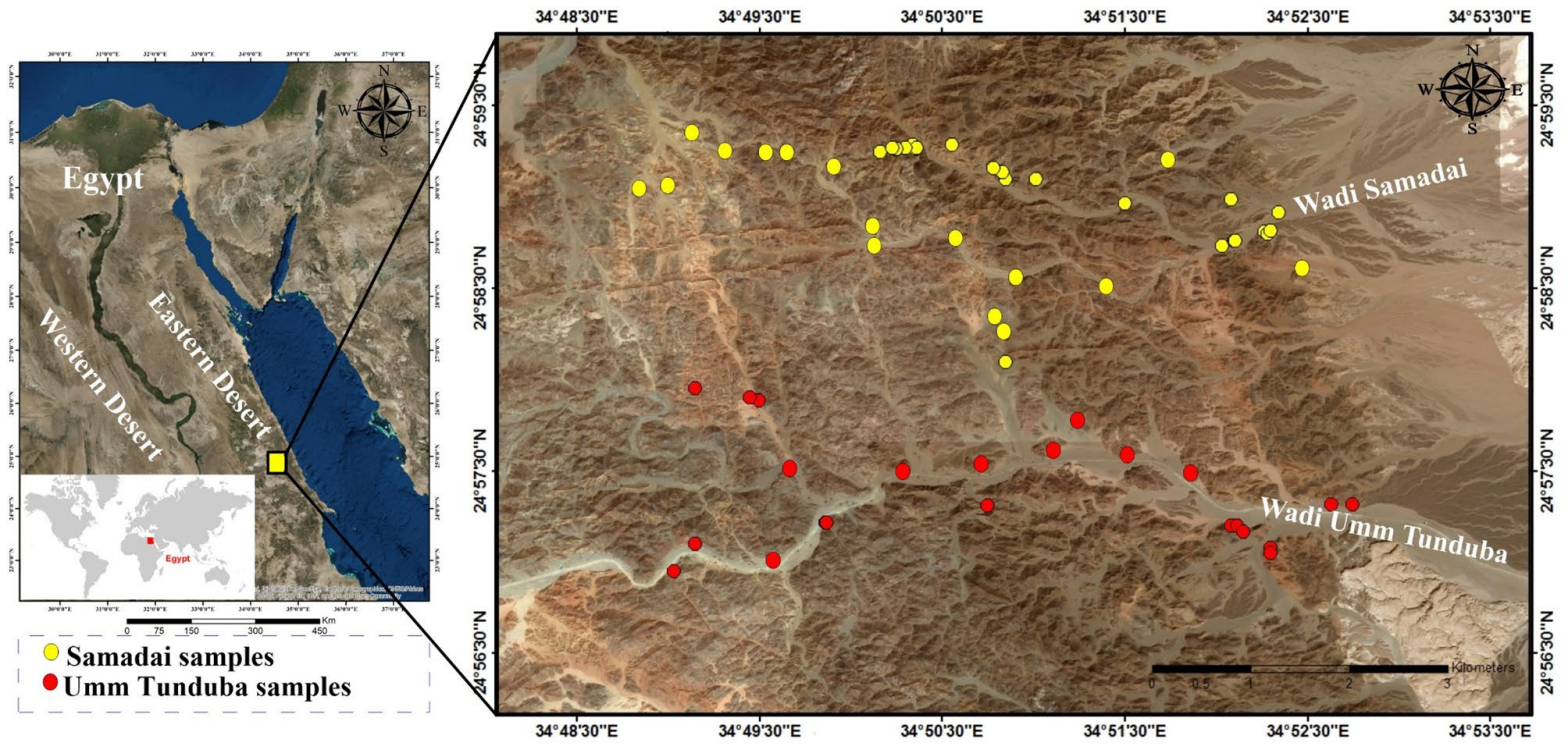
Location map showing the studied Wadi Samadai-Wadi Um Tunduba district, SED, Egypt. The map was created by ArcMap 10 (ArcGIS Enterprise, ESRI, Redlands, CA, USA).

According to previously published research^26–31^, a significant difference was observed in the distribution of rocks and their boundaries, as well as the previously published geological maps that were not accurately compatible with the nature of the area. Therefore, we made main objective of this research is to discover and delineate the main structural and lithological units of the Wadi Samadai-Wadi Um Tunduba Neoproterozoic basement, using multi-source data fusion technology, namely Landsat 9 OLI-2 multispectral and ALOS PALSAR altimetry data and a CNN-based lithological identification model. To achieve this objective, we used several statistical and spectral enhancement techniques (slope, principal components, and ratio bands) to improve and enrich the spectral information, as well as, to produce an accurate and reliable geological map. A CNN model (Fig. 1) was built to distinguish geological units based on the merged data.

## Geologic background and geomorphology

**Figure 2:**
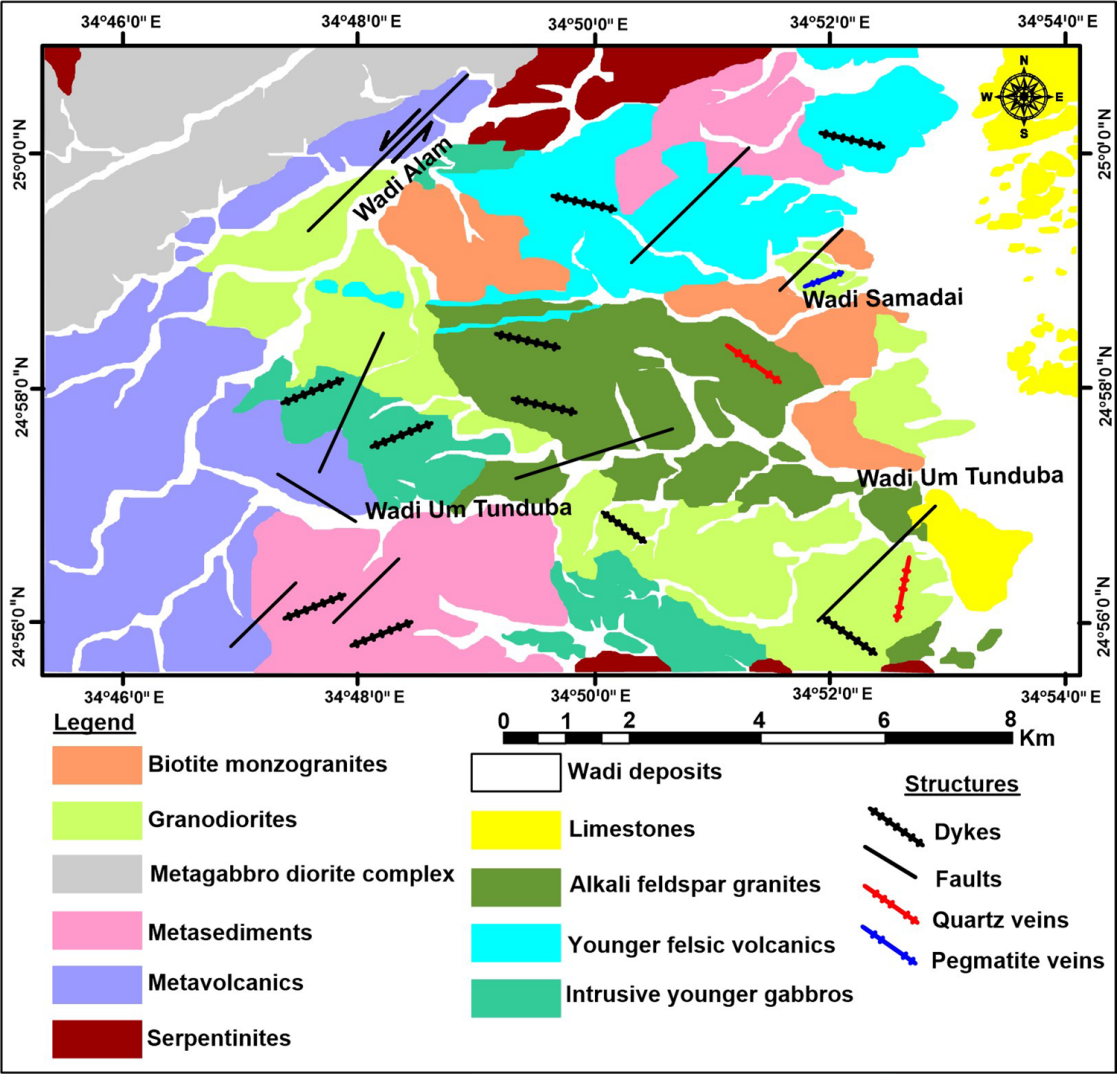
Geological map of the studied Wadi Samadai-Um Tunduba district (modified after^33^).

Wadi Samadai-Wadi Um Tunduba district is located in the southern part of the Central Eastern Desert of Egypt (CEDE) just to the north of the major low-angle thrust that marks the boundary between the Central Eastern Desert (CED) and the South Eastern Desert (SED). The SED can be partially defined by its geologic boundaries extending from Marsa Alam to the international border of Egypt at a latitude of 22° N (the Sudan line). In the northern part of the Southeastern desert is the combined tectonic boundary with the CED. The SED seems to also show infrastructure-superstructure relationships like those of the CED, but the units in the SED are somewhat older. The SED in general seems to represent a deeper level of exposure than the CED and is much less affected by Najd shearing. It is situated about 10 km Southwest of Marsa Alam city and 70 km from Abu Ghusun. The present region is approximately 283 km^2^ in area and situated between latitudes 24°56'–25°00' N and longitudes 34°46' and 34°52'30" E (Fig. 1). The geologic map of the area (Fig.2) shows master lineaments of NW and NE trends, with a prominent N–S trend in the western part of the area; the NE trend is the youngest. It was affected by the Marsa Alam Shear Zone trending from NW to SE^32^. Wadi Samadai-Um Tunduba district is covered mainly by Neoproterozoic basement rocks. The studied district is a part of the Cryogenian- Ediacaran belt encountered in the South Eastern Desert of Egypt. According to^33^, the studied district is classified according to their relative age relations into the following rock units, starting with the youngest (Fig.2).

Dismembered ophiolitic rocks (serpentinites) reach an elevation of 410 m in Gabal Samadai. The dismembered ophiolitic ultramafic rocks represent essentially by serpentinites with minor exposure's dunite, peridotite and pyroxenite which are the oldest rock unit in the study district.

Island arc assemblages are comprising essentially the metavolcanics, arc-related metasediments, and metagabbro-diorite complex. The island arc metavolcanics of are represented essentially by metabasalts, metadolerites, and meta-andesites. It occurs as low to moderate hills at the western side of the mapped area, as well as some scattered small, isolated bodies in the central and southeastern parts of the studied district. Generally, they are fine-grained rocks, but sometimes displaying porphyritic texture. The metasediments as a whole tend to be of finer to medium grain. They comprise a well-bedded repeated alternating sequence of both massive and laminated schists. The small, elongated belt of the metagabbro-diorite complex occurs either as boulders, or as medium to high-hilly masses dissected by many joint trends at Wadi Samadai (Fig.2^14^) and also exposed at the east of Wadi Um Tunduba.

Syn- to Late-tectonic stage comprises the syn- and late-tectonic granitic rocks. However, the syn-tectonic granitic rocks are represented by the tonalite–granodiorite complex. On the other hand, the late-tectonic granite is mainly represented by monzogranites and alkali feldspar granites. It is drained by Wadi Tundeba and Wadi Samadai after which the granitic mass was named. It is intruded into the surrounding metasediments, metavolcanics and metagabbros with irregular sharp contacts.

Younger felsic volcanics extruded within the metavolcanics (older), arc-related metasediments, granodiorites, and monzogranites, essentially represented by rhyolite. Intrusive young gabbros are undeformed post-tectonic plutonic rocks. They are represented by isometric un-layered, small masses intruded into the metavolcanics and the arc-related metasediments.

**Figure 3:**
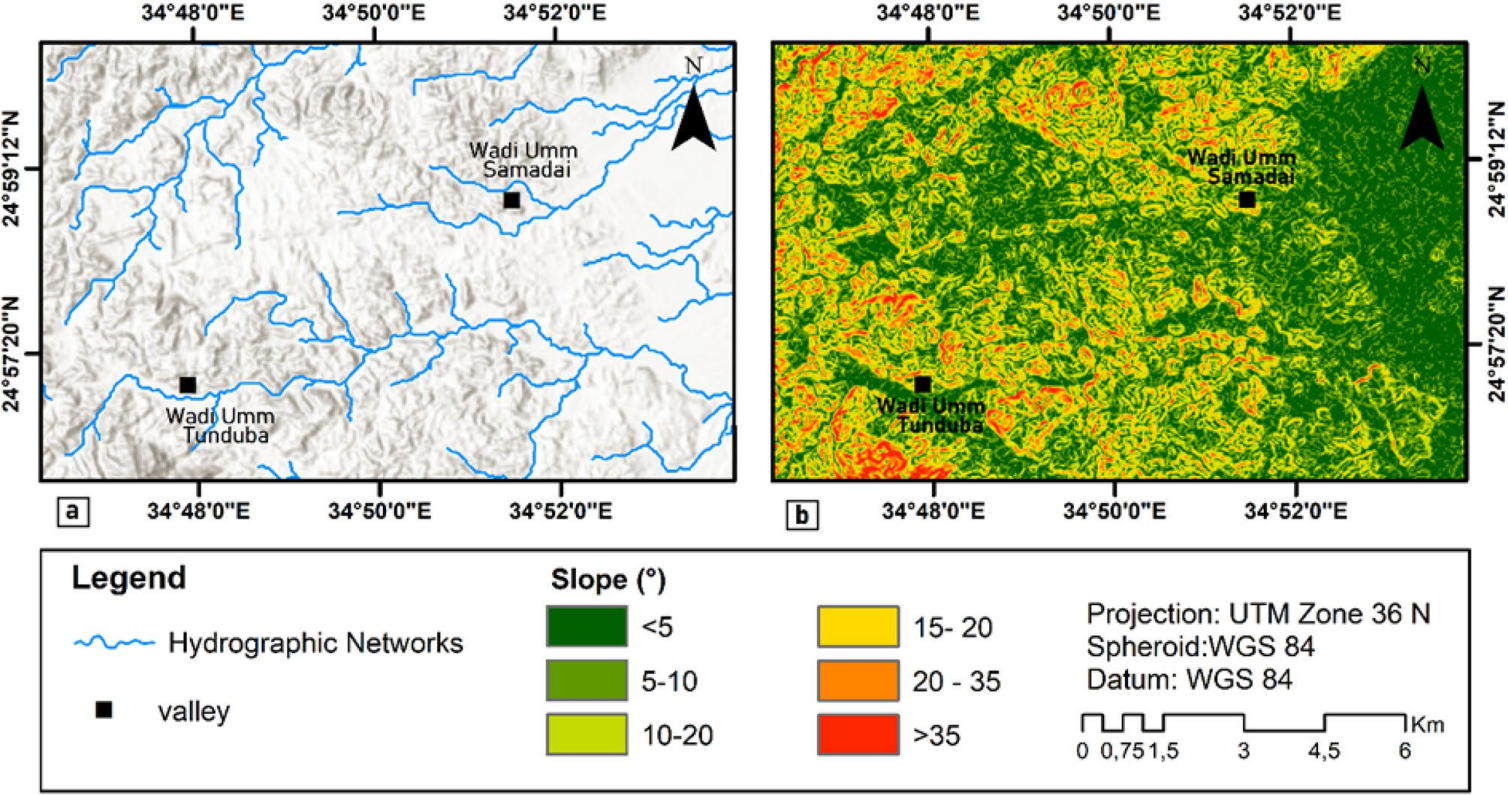
(**a**) Drainage system and (**b**) Slope model of the study district.

The relief is generally hilly and desert-like, with two valleys, Umm Tunduba and Umm Samadai, which have been shaped by wind and water erosion. The study district lies between 52 and 550 m in altitude. The highest areas rise steeply to the southwest of the study site. Geomorphologically, this district is characterized by dendritic, rectangular, and parallel drainage systems are also present in the district (Fig.3a). Due to their arid environment, the valleys are generally devoid of vegetation, except for a few drought-adapted plants, such as thorny bushes and hardy grasses. In addition, relatively steep slopes (25°–35° slope) in the northwest, south-central, north-central, and southwest, and a nearly flat plateau with a gentle slope in the east-north, east-central, and east-south (Fig. 3b) of the study district. Radial drainage can be observed in this district.

## Materials and methods

**Figure 4:**
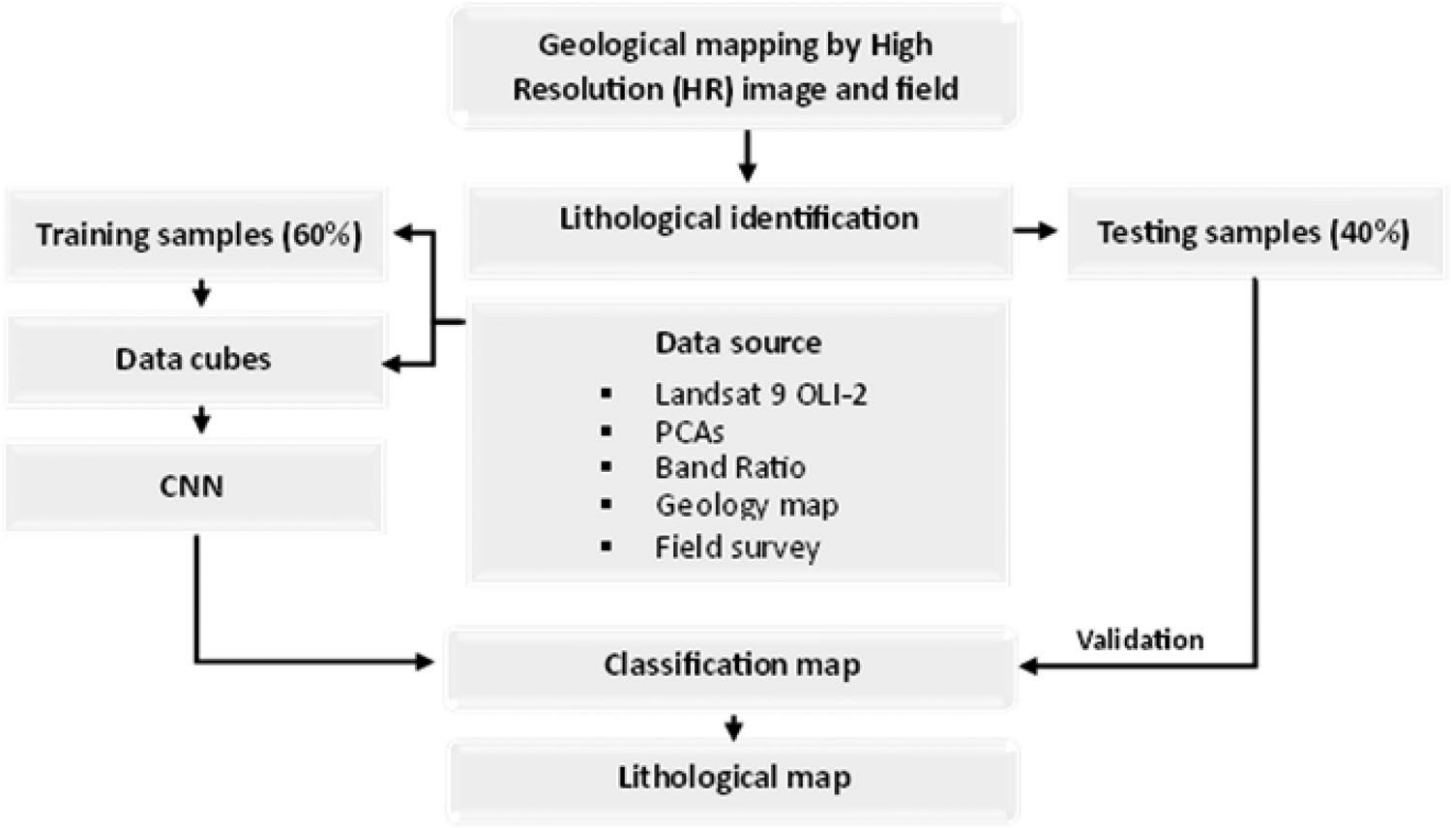
Flowchart of the study district.

In the present study, a variety of research techniques have been applied, including remote sensing data, fieldwork, laboratory work, which includes petrographical investigations (Fig. 4).

### Remote sensing technique

#### Landsat-9 image pre-processing analysis

The new multispectral remote sensing satellite Landsat-9 (Operational Land Imager 2; OLI-2), which is the most recent of the Landsat satellite heritage, was one of the satellite data sources employed in the current study. The Landsat 9 satellite carries two science instruments, the Operational Land Imager 2 (OLI-2) and the Thermal Infrared Sensor 2 (TIRS-2). The OLI-2 captures observations of the Earth’s surface in visible, near-infrared, and shortwave-infrared bands, and TIRS-2 measures thermal infrared radiation, or heat, emitted from the Earth’s surface. Both OLI and TIRS have a 5-year mission design life, although the spacecraft has 10+ years of consumables. These two devices collect data in seven multispectral bands. Coastal aerosol (B1), blue (B2), green (B3), red (B4), near-infrared (B5), shortwave infrared (SWIR) (B6), and SWIR 2 are the colors that make them up (B7). The 770 km sweep of these bands has a spatial resolution of 30 m × 30 m. They also have a 15 m × 15 m panchromatic band (B8), which is used to adapt the small-scale sensors' spatial resolution to the larger sensors' spatial resolution to extract useful information (Table 1). To perform cloud masking and quality assessments, a Cirrus band is also used (B9).

**Table 1:** Landsat 9 satellite platform, path-row details along with band types used for the analysis (https://earthexplorer.usgs.gov/).

Satellite platform	No. of bands	Band type	Wavelength range (in μm)	Spatial resolution (m)
Landsat 9-OLI (MSI) Path: 202 Row: 037	11 (8 multispectral, 1 panchromatic band, 2 thermal)	Band 1: Coastal aerosol	0.43–0.45	30
		Band 2: blue	0.45–0.51	30
		Band 3: Green	0.53–0.59	30
		Band 4: red	0.64–0.67	30
		Band 5: NIR	0.85–0.88	30
		Band 6: SWIR 1	1.57–1.65	30
		Band 7: SWIR 2	2.11–2.29	30
		Band 8: PAN	0.50–0.68	15
		Band 9: cirrus	1.36–1.38	30
		Band 10: TIRS 1	10.6–11.19	100
		Band 11: TIRS 2	11.5–12.51	100

The data used in the present study is Landsat 9 (Path 173/Row 043) downloaded from the Earth Explorer website (Earth explorer.usgs.gov) and acquired on January 19, 2022, the data is geometrically corrected and georeferenced to UTM projection (Zone 36N), Datum WGS 84 and ellipsoid. The image obtained directly from the satellite were converted from DN values to radiance.

The SAGA GIS 7.9 software suite tools were used to convert the radiance values to Top of Atmosphere (TOA) reflectance values by performing the necessary atmospheric operations. Top of Atmosphere reflectance (TOA) values by making the necessary atmospheric corrections so that spectral identification and another necessary image processing could be performed. For the facies mapping, the open-source GIS platform QGIS 3.24.3 was used. This pre-processed data was used to determine and perform the most effective ratios, PCA to produce distinct and much more interpretable images to distinguish and classify rock samples in the target area.

### Convolution neural network algorithm

Convolutional neural networks (CNNs), a significant advance in the field of machine learning algorithms, have had a profound impact on geology by revolutionising our understanding of geological landscapes^34,35^. Their ability to overcome the limitations of traditional algorithms, which focus exclusively on pixel-by-pixel classification and neglect spatial features, positions them as major transformation tools in this field.

CNNs are proving to be exceptional in the precise identification of rock formations, mineral deposits, fault lines and other geological structures^36–38^. Their hierarchical architecture, incorporating convolutional, pooling and fully connected layers, excels in feature extraction and image classification. Compared to traditional machine learning algorithms, CNNs stand out for their ability to automatically learn more complex feature representations, demonstrating robust pattern recognition and generalization skills^39,40^. Additionally, CNNs enable end-to-end learning, eliminating the need for manual feature engineering. This approach enhances the autonomy of the learning process, significantly simplifying the task of geological structure recognition.

For lithological identification, we exploit the well-established AlexNet CNN architecture^41^. As a backbone to learning high-level representations from projected images using a 10-layer structure, inspired by AlexNet with 34 convolutional layers (Conv1 to Conv34). The model architecture starts with an initial convolution layer and incorporates structured blocks to promote residual learning. This allows it to categorize many more objects. In addition, it deals with overfitting by using dropout rather than regularization^41^.

The input image size was set to 96 × 96 pixels, and ten input layers (Landsat 9 OLI-2, 30 m Aster DEM, slope, principal components, and ratio bands) were selected, a decision motivated by the search for a balance between the ability to capture the main spatial features of the target classes and the need to avoid the loss of significant spatial patterns. Experiments were carried out by varying the size of the input image windows, demonstrating that the 96 × 96 dimension proved ideal for achieving the objective. In an exploratory approach, we evaluated the use of the 15 m Landsat pixel size as a base resolution by resampling the Digital Elevation Model (DEM). This initiative reflects a proactive approach to adjusting the resolution of the data to better match the specific characteristics of the geological terrain under study.

The model training process was carried out exclusively with available data. A batch size of 50 was selected, and training was carried out over 100 epochs using a Titan RTX. The learning rate was initially set at 0.01 for the first 20 epochs, then reduced to 0.001 for the next 50 epochs, implementing a strategy for dynamically managing the learning rate as the training progressed. The final layer of the model was structured with a SoftMax classifier, characterized by an output dimension corresponding to the 11 lithological types identified in the Wadi Samadai-Wadi Um Tunduba region. This fully connected layer translates the information extracted by the model into probabilistic predictions for each lithological class, facilitating geological mapping.

## Methodology

To evaluate the CNN method adopted, we followed the procedure shown in the figure below (Fig.4). The first sections present the steps involved in the various data and their preparation. These steps were described in the previous sections.

In this study, a semi-supervised approach was used by using clustering of trained network feature maps was used. CNN algorithms are based on complex representations that characterize the features of an input image, known as "feature maps"^42^. These features take into account the distribution of data at different scales and are optimized during training. Once trained, CNN use these feature maps to make predictions based on multiple non-linear operations^36^.

Approximately 8 to 25 training samples were collected for each rock unit in the study district. Selected training samples were evaluated by using histograms, signature averaging plots and signature alarm methods to detect areas of overlap between samples collected in different bands. Supervised classification accuracy assessments were performed by using a confusion or error matrix. The pixel that was classified from the image was compared to the same geological map available and verified on the site. Reference data are selected from samples of each class by using a stratified random sampling strategy. For each class on the classification map, 8 to 25 samples are taken as reference data.

### Field work and laboratory experiments

After visual analysis of digitally processed OLI images, field investigation is used to confirm a geological map. Most of the fieldwork is devoted to verifying rock samples, and lithological differentiation. About 75 representative samples were collected from different lithological units, whereas about 59 samples are selected for petrographically examination in an attempt to establish a basis for distinguishing among the main groups.

The petrographic study includes the examination of 59 thin sections of the selected samples representing the different rock varieties in the study district. These thin sections were studied under a polarizing microscope (Optika B-353POL) combined with a digital camera at the Department of Geology, Faculty of Sciences, Al-Azhar University, Egypt.

## Results and discussion

### Spectral analysis

**Figure 5:**
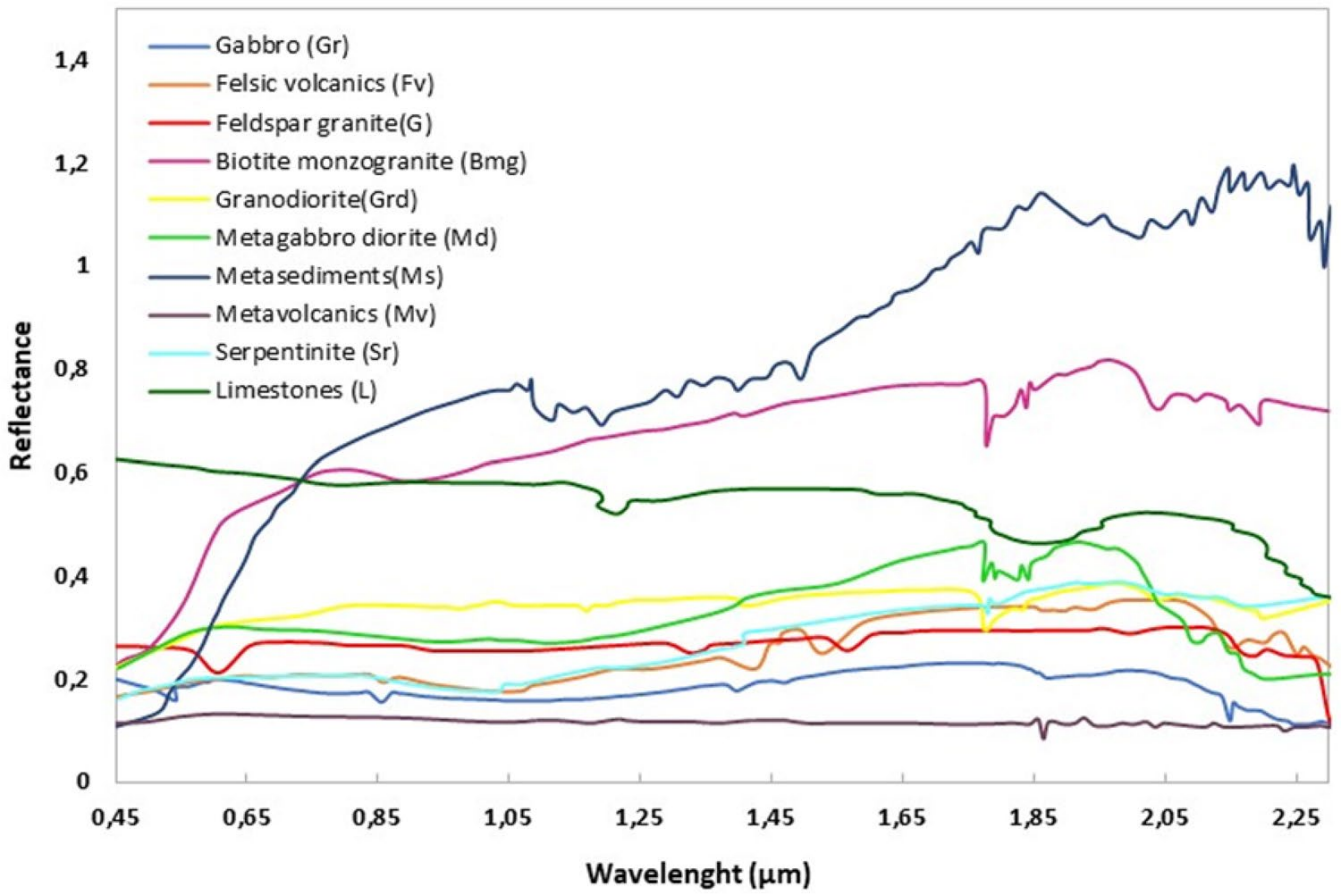
Spectral profile of different lithologies.

Spectral signature analysis is performed to identify rocks based on their spectral signature. The spectral signatures of nine rock samples were carefully selected to represent all the rock units exposed in Wadi Samadai-Wadi Um Tunduba. Analysis of reflectance profile curve results from raw and resampled data were analyzed using the Landsat-9 OLI-2 spectral specification to select the optimum band ratio for identifying and mapping the different lithologies studied. The results of these spectroscopic measurements (absorption and reflection) are mainly based on rock-forming minerals in specific wavelength parts of the electromagnetic spectrum. The resultant profile curve is illustrated in the figure below (Fig. 5).

The spectral profile measured for the gabbro samples (Fig.5) shows four absorption anomalies at 0.5–0.65 µm, 0.84–0.88 µm, and 1.5–1.6 µm. These absorption anomalies are due to the presence of gabbro-forming minerals such as augite and bytownite. Based on the above observations, gabbro shows relatively high absorption in bands 7 and 6, and relatively moderate reflectance in bands 2 and 3. Four main absorption anomalies were recorded in the spectral profile of volcanic felsic samples (Fig. 5). These absorption anomalies result from the mineral composition of this rock unit, as shown in the Fig. 5. The first occurs at 0.85–0.88 µm; this wavelength is linked to Landsat 9 band 5. The second anomaly occurs at 1.4 µm, due to the strong absorption of quartz, feldspar, amphibole, and biotite in this wavelength range. The third absorption anomaly is located around 1.5–1.6 µm; this wavelength is linked to Landsat 9 band 6. The fourth strong absorption anomaly appears at 2.2–2.3 µm, as all minerals forming felsic volcanics have strong absorption in this wavelength range. This rock shows high reflectance in bands 6 and 7.

For granite samples, three absorption anomalies were recorded on the spectral profile (Fig.5) at 0.5–0.65 µm, 1.5–1.6 µm, and 2.2–2.35 µm. These absorption anomalies are due to the presence of granite-forming minerals such as quartz, feldspar, and mica. Based on the above observations, granite shows relatively high absorption in bands 3, 5, and 6 and relatively moderate reflectances in all bands. The spectral profiles of biotite monzogranite and granodiorite are similar because their mineral composition is similar. However, biotite monzogranite shows a higher reflectance than granodiorite because the proportions of minerals forming the rock are different. The spectral profile of the biotite monzogranite and granodiorite samples shows four absorption anomalies. The first is strong at 1.6–1.8 µm, due to the presence of microcline, oligoclase and albite. The second absorption is around 1.8–1.9 µm and is due to the absorption characteristics of anorthite and orthoclase. The third and fourth absorption anomalies are located at around 2.0 µm and 2.1–2.3 µm, respectively, due to the major absorption of microcline, oligoclase, albite, and quartz (Fig.5). The spectral profile shows that biotite monzogranite and granodiorite have a high reflectance of 0.78 in the 1.6–1.8 µm range. In addition, they have high reflectance in the range (1.60–1.65 µm) covering band 6.

Four absorption anomalies were recorded for the metagabbro samples (Fig. 5). The first is located in the 1.65–1.8 µm range, due to the presence of actinolite and hornblende (Fig. 5). The second and third are located around 1.85 µm and 2.0 µm respectively, due to the presence of plagioclase, actinolite, and quartz. The fourth absorption anomaly is strong and occurs at 2.2–2.3 µm, as all the minerals forming this rock have strong absorption at this distance. Based on the above observations, metagabbros have a high reflectance in bands 6 and 7.

Several principal absorption anomalies were recorded in the spectral profile of the metasediments samples (Fig. 5). These absorption anomalies result from the mineral composition of this rock unit. The first occurs at 0.85–1.00 µm; this wavelength is linked to Landsat-8 band 5. The second anomaly occurs at 1.05–1.25 µm, due to the strong absorption of chlorite schist and mica schist in this wavelength range. The third and fourth absorption anomalies are located around 1.3 and 1.5 µm. The fifth and sixth strong absorption anomalies appear at 1.6–1.8 µm, as all minerals forming metasediments have strong absorption in this wavelength range. The others are located between 2.05 and 2.3 µm. This rock shows high reflectance in bands 5, 6, and 7.

Metavolcanics are composed of hornblende, plagioclase, actinolite, augite, hornblende, and chlorite. These minerals show strong absorption anomalies in two regions (Fig.5): the first absorption at around 1.85 µm and the second at 3–2.5 µm. On the other hand, the basic metavolcanics have a strong absorption that does not exceed 0.13 in all bands.

For serpentinite samples, two main absorption anomalies were recorded on the spectral profile (Fig. 5). The first is located in the 0.85 µm range, due to the presence of chrysotile, lizardite, and antigorite. The second absorption anomaly occurs in the 1.7–1.85 µm range, due to the strong absorption of kaolinite, chrysotile, lizardite, and antigorite. Serpentinites have relatively high absorption in near-infrared band 5 (0.85–0.88 µm) and mid-infrared band 7 (1.5–1.66 µm). On the other hand, serpentinites show high reflectance in bands 5 and 7.

The spectral profile measured for limestone samples (Fig.5) shows several absorption anomalies at 1.1 µm, 1.2 µm, 1.7 µm, 1.76 µm, 1.9 µm, 2.1 µm, 2.15 µm, and 2.2 µm. These absorption anomalies are due to the presence of limestone-forming minerals such as calcite and aragonite. Based on the above observations, limestone shows relatively high absorption in bands 6 and 7, and relatively high reflectance in all bands.

### Landsat-9 image processing analysis

#### Principal component analysis (PCA)

Principal component analysis (PCA), which reduces the variance of the original bands and shows correlations in the raw remote sensing data, which is very useful for identifying the lithological characteristics of rocks and minerals on the basis of their spectral properties^43–46^. The first PC1 has the highest variance, 96.12% of all variance data and includes an equal mix of all bands; however, band 5 has more influence (higher eigenvalue of 0.325768, followed by SWIR1, SWIR2, and near infrared bands, respectively). It includes the most information in the input bands and can therefore be used to illustrate lithological and structural features. On the other hand, the last PC contains the least information from the input bands, accounting for 0.008% of the total variance. PC2 represents 2.92% of the total variance, perpendicular to PC1, and shows the spectral variations between the visible data, with the highest positive charge (0.18) for band 1 and the highest negative charge (− 0.94) for band 2.

The first three PCAs generally comprise more than 99.5% of the total variance (Table 2); the second and third PCAs can detect surface features that were not initially distinctive due to the high similarity of the original data. In addition, PC4 has a high positive value 0.223510 in band 1 and a low negative value − 0.7959 in band 4; thus, PC4 is useful for discriminating between rock units. However, visual inspection of the upper PCs revealed important information relating to the distribution of lithological units. However, in the current work, lithological discrimination and the delineation of structural features were achieved extremely successfully by using Landsat-9 (OLI-2) pictures in grayscale VNIR-SWIR bands.

**Table 2:** Eigenvalues and variances % for the PCA of Landsat-9 (OLI-2) bands.

PC no	Band1	Band2	Band3	Band4	Band5	Band6	Eigenvalues	Variance
PC1	0.611502	0.187288	−0.510446	0.223510	0.325768	0.262200	0.03433395	96.1296150
PC2	0.187288	−0.948238	−0.516111	−0.094177	−0.601876	−0.360007	0.00104646	2.9299339
PC3	−0.510446	−0.516111	0.770400	−0.311976	0.030552	0.090168	0.00021973	0.6152120
PC4	0.223510	−0.094177	−0.311976	−0.795954	0.097187	0.118812	0.00009949	0.2785608
PC5	0.325768	−0.601876	0.030552	0.097187	−0.846079	0.019577	0.00001380	0.0386502
PC6	0.262200	−0.360007	0.090168	0.118812	0.019577	0.856792	0.00000287	0.0080281

**Figure 6:**
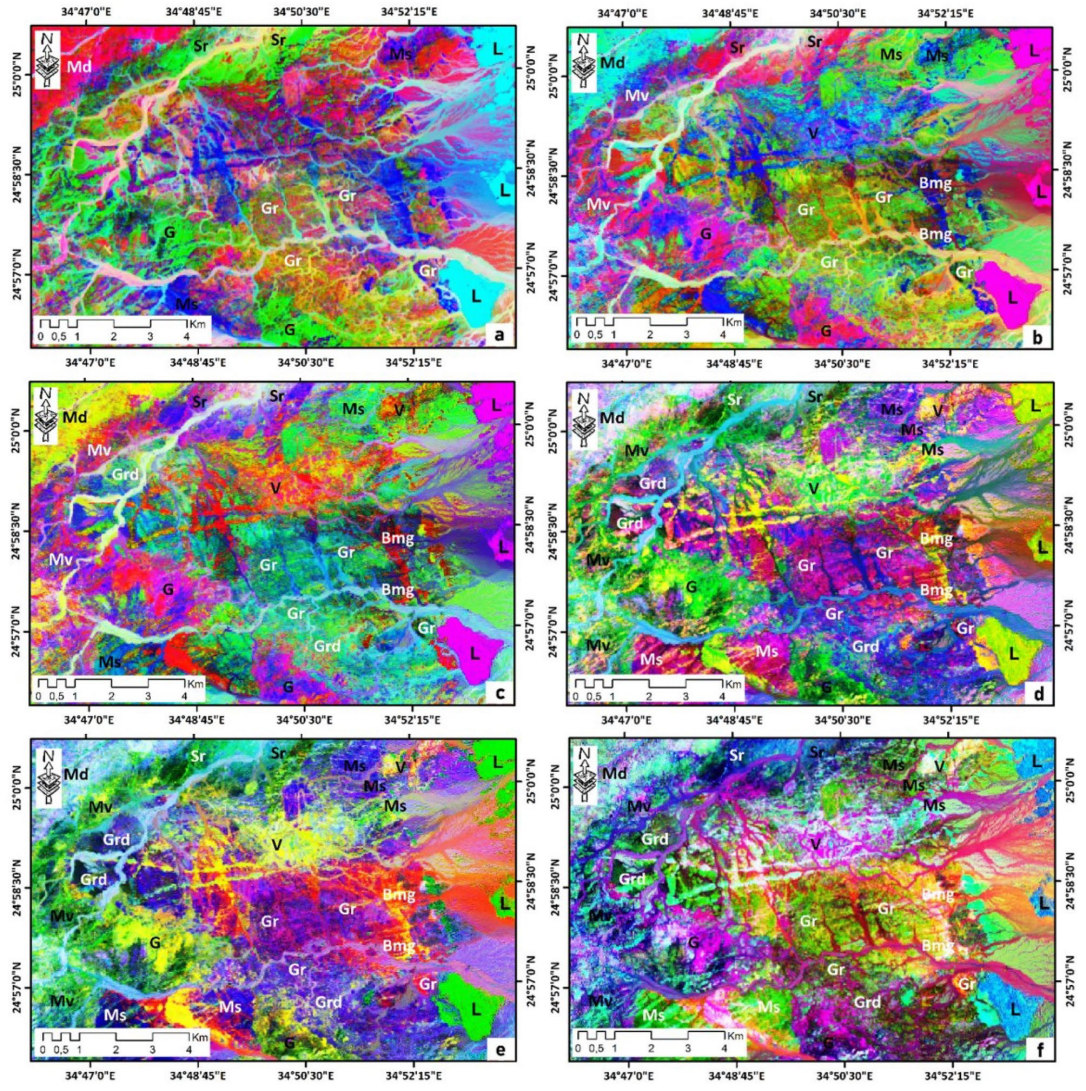
Principal Component analysis of Landsat-9 (OLI-2) image for of Wadi Samadai-Wadi Um Tunduba district. (**a**) (PC3, PC2, and PC1), (**b**) (PC2, PC3, and PC4), (**c**) (PC4, PC3, and PC2), (**d**) (PC5, PC4, and PC3), (**e**) (PC6, PC4, and PC3), (**f**) (PC6, PC5, and PC4) in RGB, respectively.

The combination of PC4, PC3, and PC2 in RGB is the best for lithological identification. In this PC combination, almost all lithological units have been classified. This PC composite is excellent for differentiating metagabbro-diorite complex in yellow-green, limestones in red-purple, metavolcanics in purple, metasediments in green, younger felsic volcanics in fire-red, and biotite monzogranites in blue-red. In this PC composite, the alkali feldspar granites are characterized by a pine-green color (Fig. 6c). Other PCA composite images (PC3, PC2, and PC1), (PC2, PC3, and PC4), (PC5, PC4, and PC3), (PC6, PC4, and PC3), (PC6, PC5, and PC4) were selected for lithological mapping.

On the PCA composite image (PC3, PC2, and PC1; Fig.6a), four lithological units were well discriminated: limestones (light blue), alkali feldspar granites (green), metagabbro-diorite complex (pink) and metasediments (light pink). In contrast, on the PCA composite image (PC2, PC3, and PC4; Fig.6b), eight lithologies: limestones (red-purple), alkali feldspar granites (green-Veronese), metagabbro-diorite complex (light blue), and metasediments (light green), younger felsic volcanics (blue), biotite monzogranites (blue-green), gabbros (pink), and metavolcanics (pink-blue).

The other PC composite images presented (PC3, PC2, and PC1), (PC3, PC2, and PC1), and (PC3, PC2, and PC1) in Fig. 6d–f respectively show almost all lithological units, ten units were well discriminated.

#### Band ratio images

The band ratio method is very important for highlighting specific materials that cannot be seen in raw remote sensing data^47,48^. Band ratio is useful for recognizing geological features. Based on the analysis of spectral reflectance curves, the study proposed four Landsat-9 (OLI-2) band ratio composite images: (6/5, 4/1, and 2/1), (4/2, 6/7, and 5/6), (5/4, 3/2, and 2/1) and (3/2, 5/6, and 4/6) in RGB.

**Figure 7:**
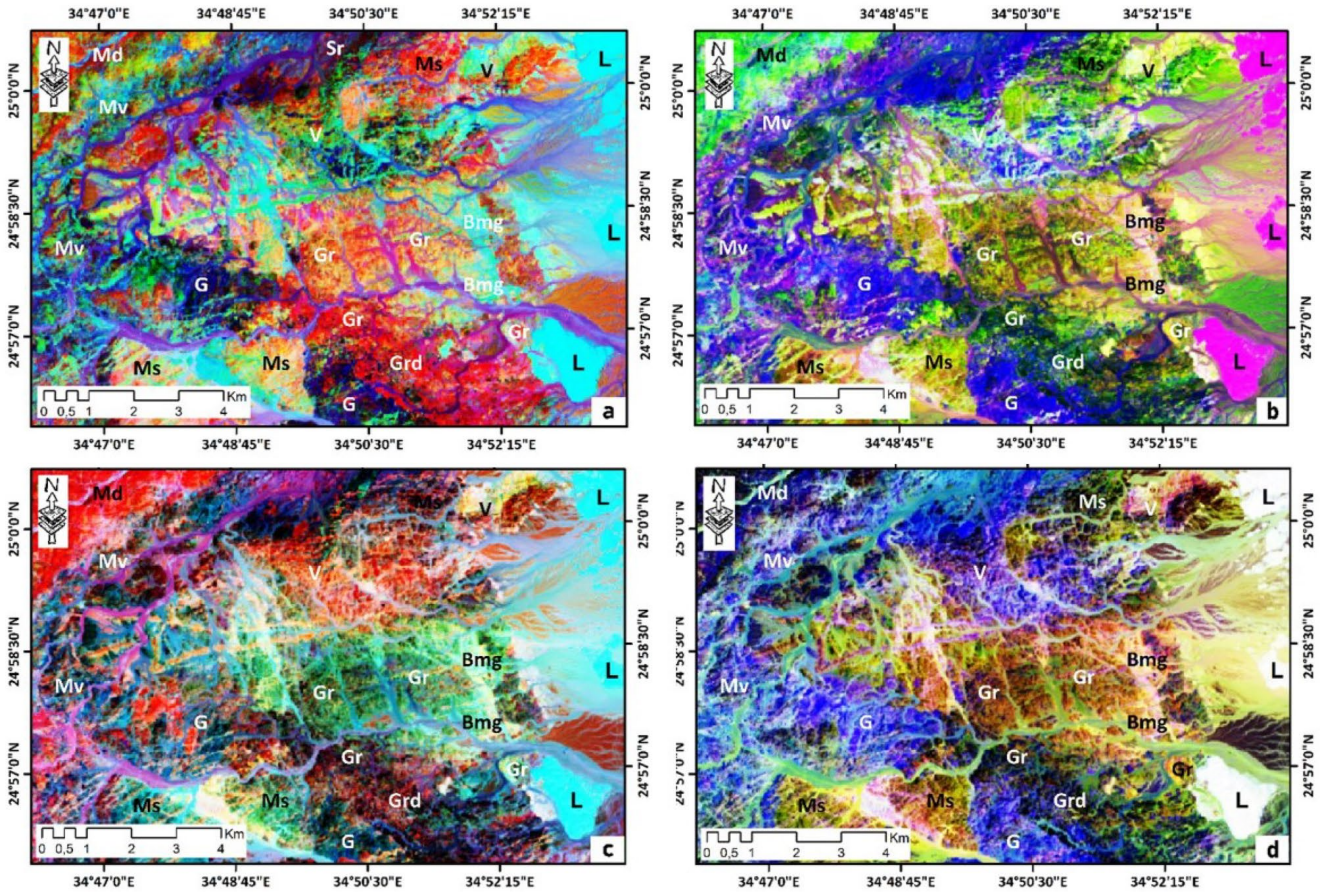
Landsat-9 (OLI-2) band ratio images covering the of Wadi Samadai-Wadi Um Tunduba district. (**a**) (6/5, 4/3, and 2/1), (**b**) (4/2, 6/7, and 5/6), (**c**) (5/4, 3/2, and 2/1), and (**d**) (3/2, 5/6, and 4/6) in RGB, respectively.

Detailed inspection of these ratio combinations shows the particular characteristics of certain rock units. The 6/5 ratio is useful for mapping rock-forming iron minerals, as these minerals have a high reflectance in this ratio. In the band ratio composite image (6/5, 4/3, and 2/1); (Fig. 7a), limestones are cyan, while granites are orange, metagabbro-diorite complex is brown, gabbros is blue, metavolcanics are green, and granodiorites is red. On the other hand, the composite band ratio image (4/2, 6/7, and 5/6; Fig. 7b) was the most discriminating of the four combinations. The 4/2 ratio is used to map iron oxides, the metagabbro-diorite complex was well detected on this combination (light green), metavolcanics in blue, limestones in light purple, metasediments in green, and biotite monzogranites in beige.

On the other two band ratio combinations (5/4, 3/2, and 2/1) and (3/2, 5/6, and 4/6), shown in Fig. 7c, d respectively. More lithological units were well discriminated. For the first combination (Fig. 7c), the metagabbro-diorite complex was well detected in this combination (red), metavolcanics in violet-blue, limestones in cyan-blue, metasediments in green, and biotite monzogranites in beige, granites in green, volcanic felsic in yellow, and granodiorite in dark red. By contrast, for the second combination (3/2, 5/6, and 4/6), the metagabbro-diorite complex appears in dark blue, limestones in white, biotite monzogranite in violet, granodiorite in green-blue, and metasediments in olive green.

### Classification results

**Figure 8:**
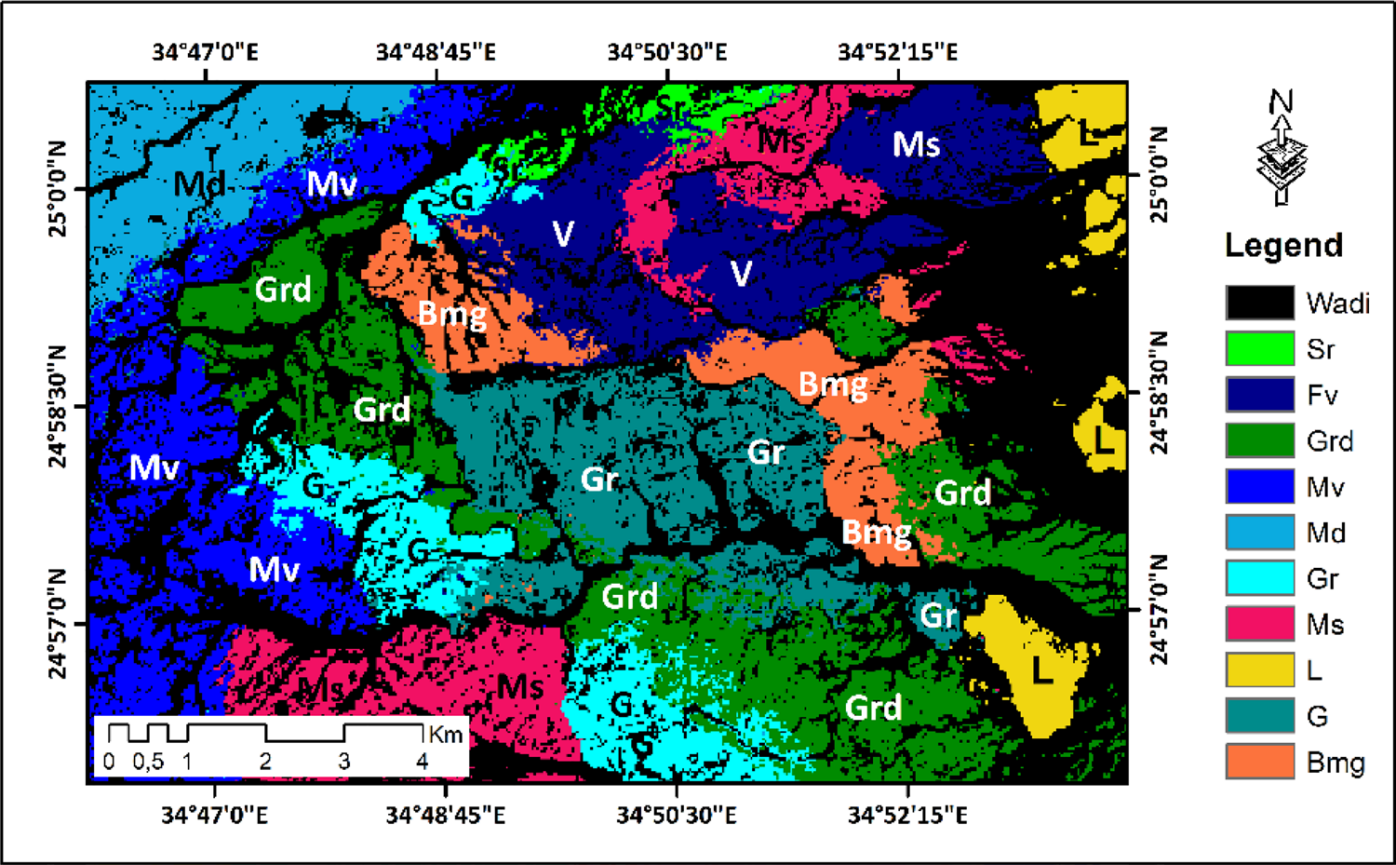
Landsat-9 (OLI-2) classified image for Wadi Samadai-Wadi Um Tunduba district.

The results obtained by our methodology enable us to produce a lithological map by forming an evolutionary neural network by using the various detailed data (Fig. 8). The classified map below shows ten lithological units.

To assess the resulting classified map, the accuracy evaluation was calculated for all resampling methodological combinations. For this survey, test categories were formed for the evaluation of classification accuracy. Pixels were selected at random. Two thousand and five hundred sample points were chosen (Table 3). We used overall accuracy, user and producer accuracy, and the Kappa coefficient (standard) for accuracy assessment.

**Table 3:** Classification accuracy assessment.

Lithoclass	Sr	Fv	Grd	Mv	Md	Gr	Ms		L	G	Bmg
No of test pixels	60	310	340	251	270	367	230		186	329	157
Producer’s accuracy (%)	93.87	89.41	99.12	98.9	99.17	97.13	98.97		96.45	97.16	98.77
User’s accuracy (%)	93.75	94.44	92.86	92.25	92.36	91.31	92.94		94.59	92.86	93.94
Kappa coefficient	0.93	0.93	0.91	0.91	0.90	0.91	0.92		0.94	0.92	0.93
Overall accuracy (%)	95.27					Overall kappa	0.94				

Abbreviation showing in Fig. 5.

The classified map (Fig. 8) shows good agreement between the thematic map generated from the Landsat-9 image (OLI-2) and the reference data. The accuracy of the lithological map was assessed by using validation pixels, and the results summarized by using a confusion matrix (Table 3). According to accuracy assessment techniques, the results indicate an overall accuracy of 95.27%. The Kappa coefficient value of 0.94 indicates very good agreement between image-generated thematic maps and reference data.

### Field verification

For more confidence, the above description in the remote sensing part for each exposed rock unit, the findings of a thorough field verification process were used to assess and confirm the distribution data obtained.

#### Metamorphic assemblages

**Figure 9:**
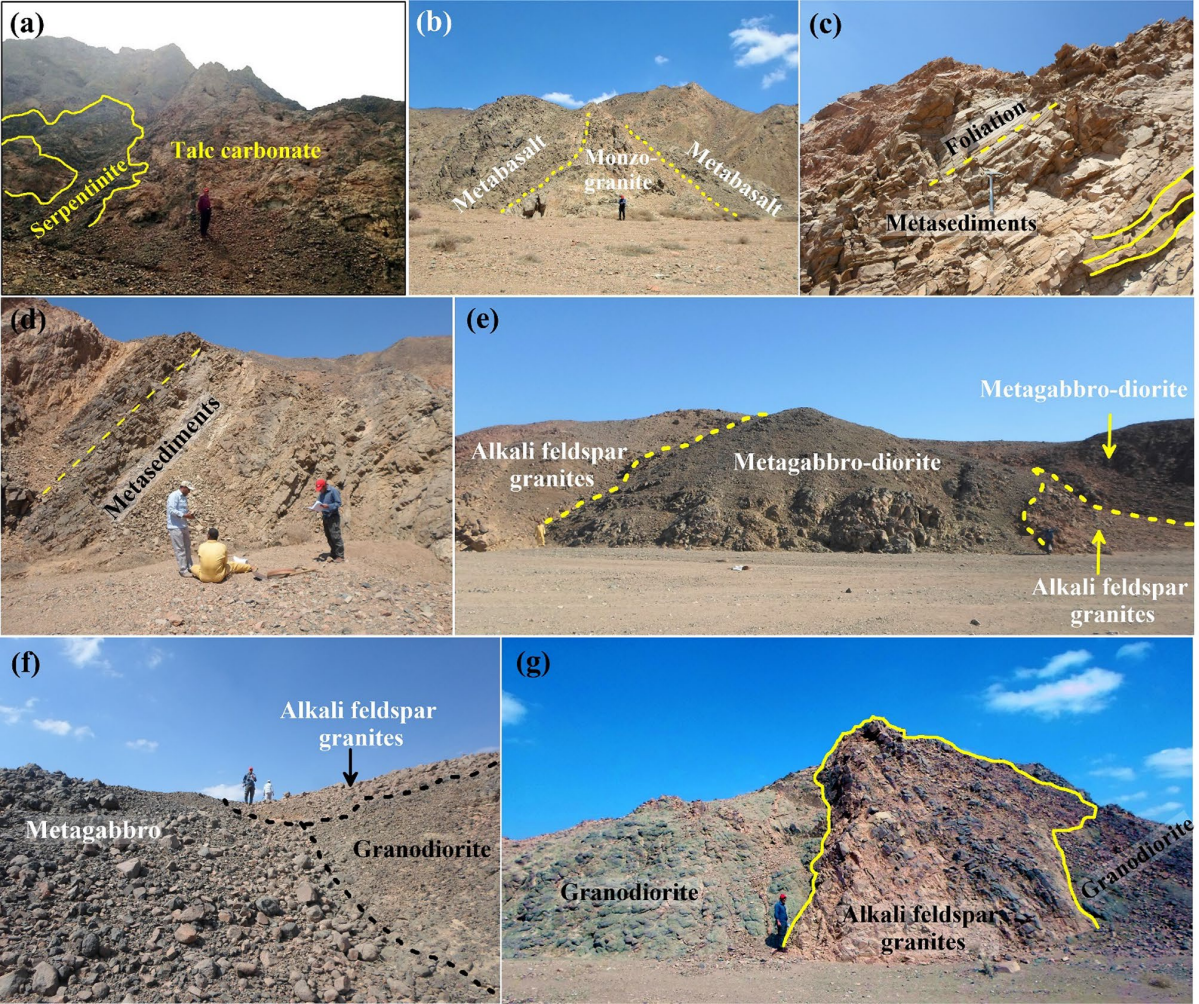
Field observation showing the main geological features at Samadai-Um Tunduba district. (**a**) Highly sheared serpentinite associated to talc-carbonates, Samadai area, looking E. (**b**) Metavolcanics intruded by biotite monzogranites, Samadai area, looking NE. (**c**) Arc-related metasediments exhibits well-developed foliation, Um Tunduba area, looking SW. (**d**) Planer structures of the arc-related metasediments, Samadai area, looking SW. (**e**) Alkali feldspar granites intruded into the metagabbro-diorite complex, Um Tunduba area, looking NE. (**f**) Sharp contact between metagabbro, granodiorites, and alkali feldspar granites, Samadai, Looking SW. (**g**) Clear body intrusion of alkali feldspar granites penetrating the granodiorite, Um Tunduba area, looking NW.

Serpentinites considers the oldest rock unit in the study district, which are dismembered ophiolitic rock units. These serpentinites are moderate to high mountains, as well as vary in colors from greenish grey to dark green, brownish, and violet. It is distributed in the northern portion of Wadi Samadai and as small mass in the south-western part at the end of Wadi Tunduba (Fig. 9a). There are extremely few talc-carbonate rocks in the Gabal Samadai region, and they only appear in shearing zones as a result of CO_2_ metasomatism of serpentinites (Fig. 9a).

Metavolcanics mainly occurred in the western sector of the study district. They are highly sheared, jointed, and composed essentially of metabasalts. Metabasalts are intruded by biotite monzogranites (Fig. 9b).

Arc-related metasediments are commonly exposed in the northern part of Wadi Samadai (Fig. 9c) and southern part of Wadi Um Tunduba. These rocks are mainly dark-colored, massive to strongly foliated, chiefly mafic, graded welded crystal tuffs. They are varies in grain sizes from fine- to medium-grained and characterized by planer structures (Fig. 9d).

The metagabbro-diorite complex represents the younger phase of the island arc assemblages. They are exposed on the northwestern part of the study district. It occurs as boulders, medium to high hilly masses dissected by many joint trends at Wadi Samadai (Fig. 9e). These rocks intruded by granodiorite and alkali feldspar granites (Fig. 9f).

### Magmatic assemblages

**Figure 10:**
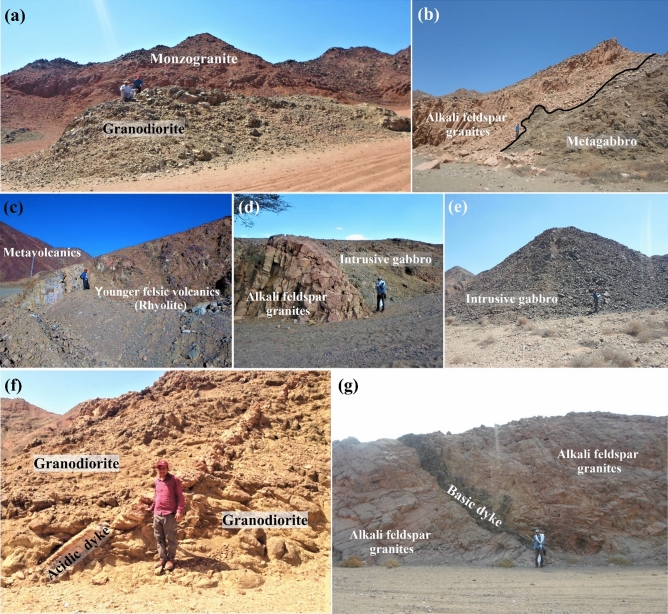
Field photographs showing the main rock units in Samadai-Um Tunduba district. (**a**) Low hills of monzogranite intruded within granodiorite, Samadai area, looking NW. (**b**) Sharp contact between alkali feldspar granites and metagabbros, Um Tunduba area, looking NE. (**c**) Younger felsic volcanics (rhyolite) extruded within metavolcanics, Um Tunduba, looking SE. (**d**) Sharp contact between alkali feldspar granites and intrusive young gabbros, Um Tunduba, looking SE. (**e**) Small masses of intrusive young gabbros, Samadai area, looking SW. (**f**) Acidic dykes cutting cross the granodiorites, Samadai area, looking SW. (**g**) Basic dyke extruded within the alkali feldspar granites, Samadai area looking SE.

Syn- to late-tectonic granites: the syn-tectonic granitic rocks are represented by the granodiorites, while the late-tectonic granite includes biotite monzogranites and alkali feldspar granites. Samadai granitic mass is situated in central, southeast of the mapped area. These granitic masses belonged to syn to late-tectonic stages of pan-African movement. Granodiorites often occur as massive rocks, grey color (Fig. 9g). Sometimes, they are highly weathered and deformed especially near the contact with the older rock units. Um Tunduba granodiorite intruded by alkali feldspar granites (Fig. 9g). The biotite monzogranite is represented by a high body, pinkish-grey and pinkish red in color, medium- to coarse-grained, highly weathered, and jointed (Fig. 10a). Alkali feldspar granites are pink or red in color, medium- to coarse-grained, and are extensively weathered along the joints. They are exposed in the central and eastern parts of the mapped area. These rocks are extensively fractured and jointed (Fig. 2) to form huge blocks. Alkali feldspar granites enclose basic enclaves and xenoliths of metagabbros and granodiorites, which are irregularly distributed throughout the granite pluton. The alkali feldspar granites intruded within metagabbros, granodiorites, and intrusive gabbro, with sharp contact (Fig. 10b).

Younger felsic volcanics are widespread in the study district, especially in the northeastern part of Wadi Samadai and in the western part of the mapped area (Fig.2). They are represent by rhyolite. Rhyolite is occurring as small exposure encountered in the western part at the end of Wadi Um Tunduba (Fig. 10c). It is characterized by light grey color, fine- to very fine-grained.

Intrusive young gabbros are highly weathered, characterized by abundant content of pyroxene, calcic plagioclase, and hornblende minerals. Wadi Samadai alkali feldspar granites are intruded directly by small plug-like masses of gabbro, which simulate the young intrusive gabbros (Fig. 10d, e).

Dykes and veins: Wadi Samadai-Wadi Um Tunduba district is traversed by numerous dyke swarms, which consequently crossed all the pre-existing rock units. Most of these dykes have NE–SW trend, less often they have NW-SE. The thickness ranges from centimeters to more than 1m. These dykes are represented by acidic and basic dykes. Acidic dykes are represented by granitic and rhyolitic dykes (Fig. 10f). The granitic dykes are pink to red in color, fine-grained with thickness ranging from 25 cm to 1.5 m. On the other hand, the rhyolitic dykes are light grey and red in color, fine-grained with thickness up to 4 m. Basic dykes are represented by dolerites and basalts. They are

characterized by the less weathered and dark green in color with length ranges from 1 to 4 m (Fig. 10g).

### Petrography

**Figure 11:**
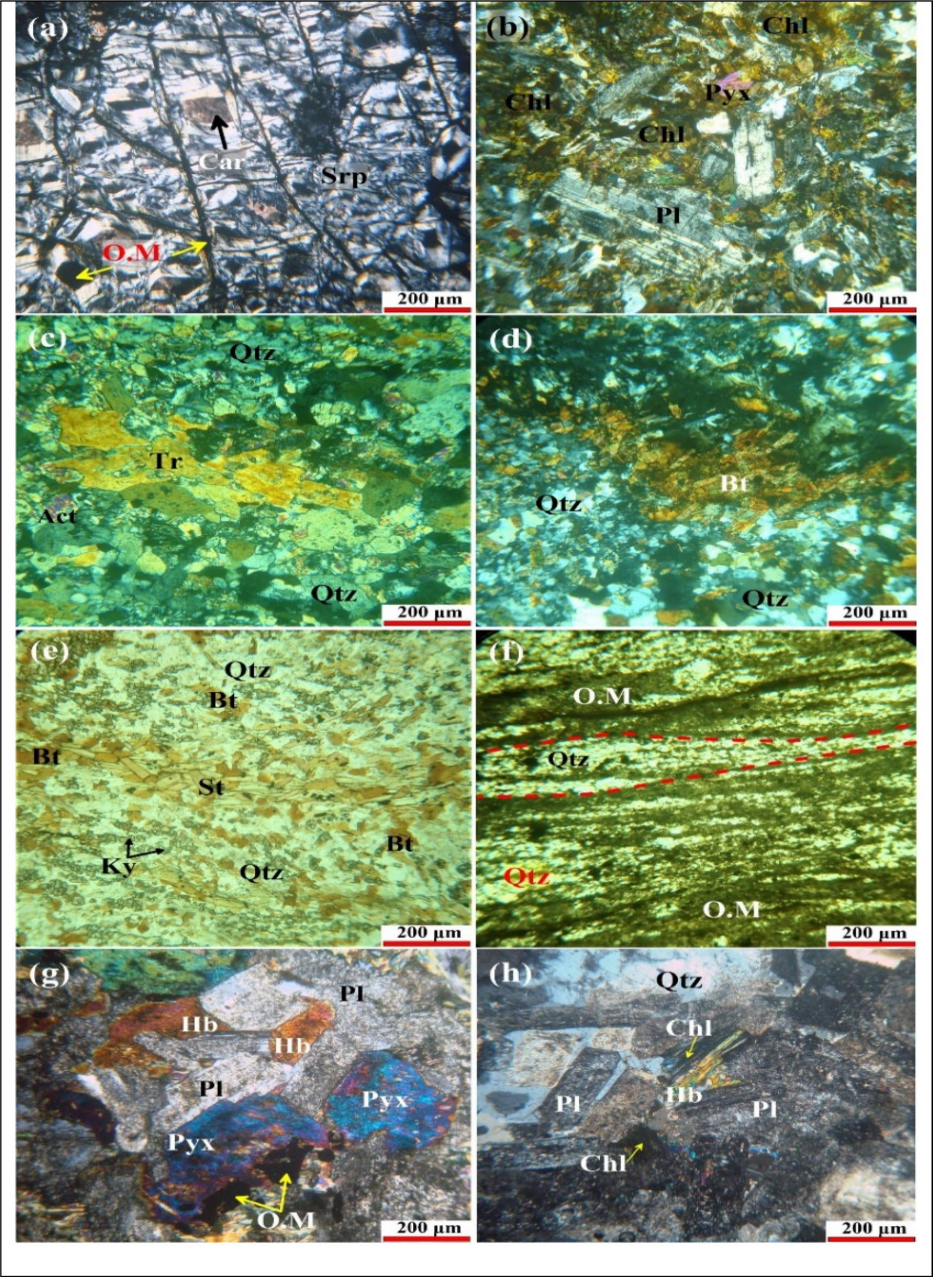
Photomicrographs showing the main petrographic features of Samadai-Um Tunduba rock units. (**a**) Well-developed ribbon texture within serpentinite dissected by opaque microveinlets (CN). (**b**) Ophitoblastic and diabasic textures in metabasalts (CN). (**c**) Prismatic crystals of tremolite and actinolite in tremolite schists (CN). (**d**) Aggregates of biotite crystals within staurolite-kyanite-biotite schists (CN). (**e**) Oriented biotite (Bt) flakes interstitial between quartz (Qtz), staurolite (St), and kyanite (Ky) in staurolite-kyanite-biotite schists (PPL). (**f**) Mylonitic texture as well as the mafic and felsic minerals exhibits the foliated texture in the mylonitic schists (PPL). (**g**) Coarse-grains of plagioclase (Pl), pyroxene (Pyx), and hornblende (Hb) in metagabbros (CN). (**h**) Highly saussuritized plagioclase (Pl) crystals and hornblende altered to chlorite in diorites (CN).

Serpentinites are mainly composed of serpentine minerals (antigorite-chrysotile) with secondary talc carbonates as well as accessory opaques (Fig. 11a). Opaque minerals are mainly magnetite and chromite, which occurs as euhedral crystals of bloody color or as dark grains. It characterized by mesh and ribbon textures.

Metavolcanics are represented mainly by metabasalts. These rocks are composed of plagioclase and pyroxene as well as some secondary minerals such as actinolite, chlorite, and carbonate minerals. Sometimes, they exhibit ophitoblastic and diabasic texture (Fig. 11b).

Arc-related metasediments are represented by schists. They are characterized by medium-grade metamorphic rocks with sheet-like grains in a preferred orientation. According to the mineral composition and grade of metamorphism, the schists are divided into three types: tremolite schists, staurolite–kyanite–biotite schists, and mylonitic schists.

Tremolite schists are composed essentially of amphiboles, plagioclase, and quartz as well as veinlets of quartz and plagioclase crosscutting the foliation. Amphiboles represented by tremolite and actinolite (Fig. 11c). Staurolite–kyanite–biotite schists are dominated by staurolite, kyanite, and biotite interlayered with quartz and feldspar (Fig. 11d). It is a fine- to medium-grained schistose texture. Prismatic staurolite and kyanite are preferred orientations, which show nematoblastic texture (Fig. 11e). Mylonitize schists formed by ductile deformation during intense shearing encountered during folding and faulting, a process termed (cataclastic or dynamic metamorphism). These grains are crushed and characterized by porphyroclastic texture. It is mainly composed of plagioclase, quartz, and they have the same foliation association with the opaque minerals (Fig. 11f).

Metagabbros are medium- to coarse-grained, granular, hypidiomorphic and exhibit ophitic and subophitic textures (Fig. 11g). They are composed mainly of calcic plagioclase ranging from bytownite to labradorite, pyroxene, and hornblende. Quartz diorites exhibit equigranular and hypidiomorphic textures. They are composed essentially of plagioclase, hornblende, and quartz (Fig. 11h). Biotite, chlorite, and epidote occur as secondary minerals associated with accessory minerals, which are represented by zircon and opaques.

**Figure 12:**
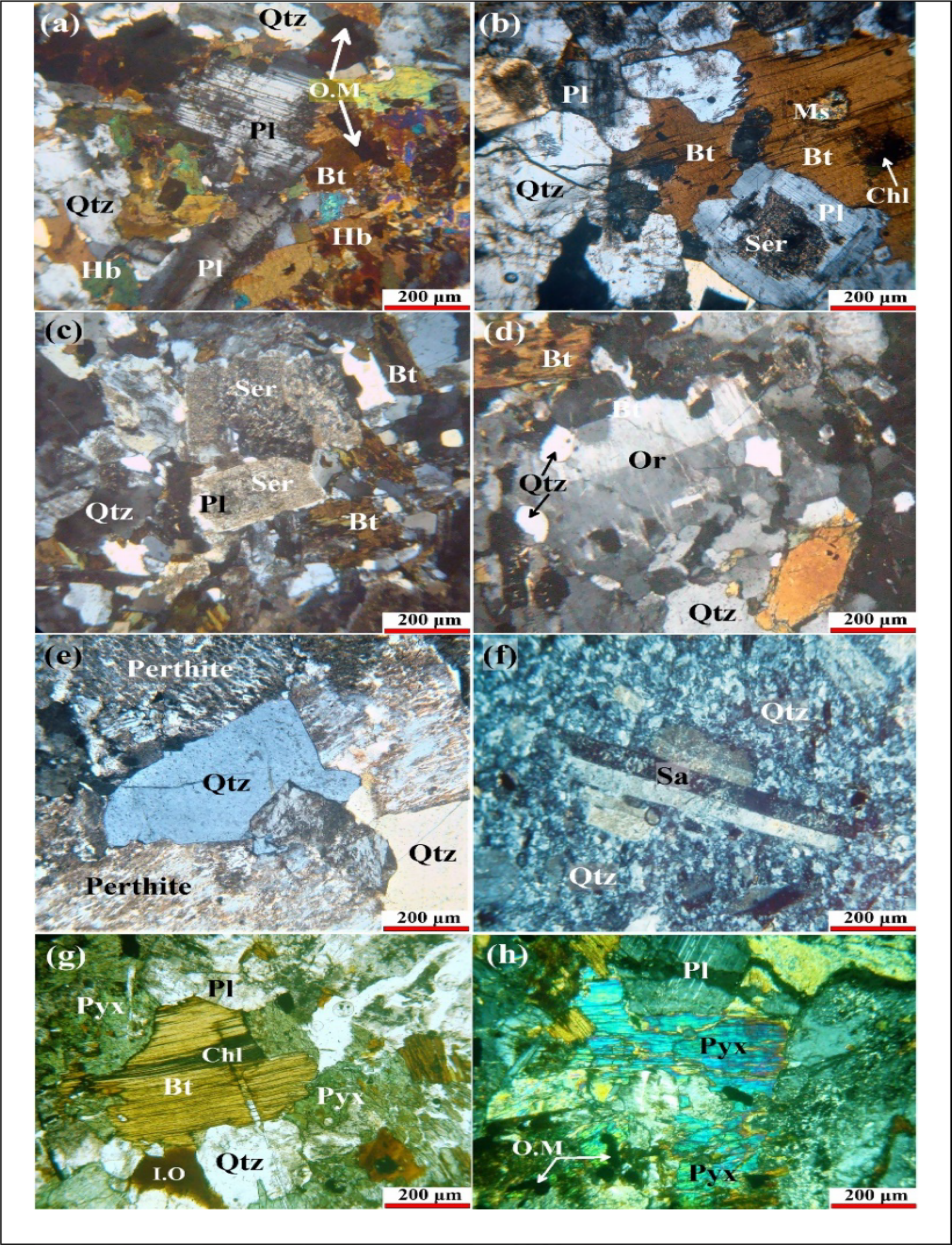
Photomicrographs showing some petrographic features of Samadai-Um Tunduba rock units. (**a**) Accumulation of mafic minerals (biotite: Bt–hornblende: Hb–opaque mineral: OM) with felsic minerals (quartz: Qtz–plagioclase: Pl) in granodiorites (CN). (**b**) Replacement of plagioclase (Pl) by sericite (Ser) is associated with alteration of biotite to muscovite (Ms) in granodiorites (CN). (**c**) Hypidiomorphic texture including highly altered of plagioclase (Pl) to sericite (Ser) crystals in monzogranites (CN). (**d**) Simple twinning of orthoclase (Or) associated with perthitic and poikilitic textures in monzogranites (CN). (**e**) Coarse grains exhibited quartz (Qtz) and perthitic texture in alkali feldspar granites (CN). (**f**) Sanidine phenocrysts (Sa) forming porphyritic texture in rhyolites (CN). (**g**) Pyroxene (Pyx) encloses the plagioclase (Pl) and biotite altered to chlorite in intrusive gabbros (CN). (**h**) Coarse grains of pyroxene (Pyx) associated to opaque minerals forming subophitic texture in intrusive gabbros (CN).

The granodiorite is medium- to coarse-grained rocks, often equigranular texture (Fig. 12a). They are composed essentially of altered plagioclase, quartz, and potash feldspars with minor amounts of biotite and hornblende (Fig. 12b). Biotite monzogranites are medium- to coarse-grained and show hypidiomorphic granular and poikilitic texture (Fig. 12c, d). They are composed essentially of quartz, orthoclase, plagioclase, biotite, and hornblende. Sericite, chlorite, epidote, and kaolinite are secondary minerals. Alkali feldspar granites are characterized by perthitic texture and consist mainly of potash feldspar, quartz, plagioclase, and biotite. Potash feldspar is the main constituent and is represented by orthoclase- and microcline-microperthites (Fig. 12e).

The studied rhyolite is a fine-grained rock and has often porphyritic and spherulitic textures (Fig. 12f). It is composed essentially phenocrysts of quartz, sanidine, and plagioclase in addition to a minor amount of opaques crystals set in a fine-grained groundmass. The intrusive young gabbro consists essentially of plagioclase, pyroxene, and hornblende. Biotite is less common, and opaques are the main accessory minerals. These rocks exhibit ophitic and subophitic textures (Fig. 12g, h).

## Conclusions

1. Wadi Samadai-Wadi Um Tunduba district is covered by basement rocks of Precambrian age nonconformably overlain by sedimentary rocks of Phanerozoic age in southern portion. This district is occurring between latitudes 24°56'–25°00' N and Longitudes 34°46'–34°52'30" E.
2. According to the field and petrographical studies the studied district is occupied by metamorphic rocks that dominated by serpentinites, metavolcanics, arc-related metasediments, metagabbro-diorite complex. While magmatic assemblages are include granodiorites, biotite monzogranites, alkali feldspar granites, younger felsic volcanics (rhyolites), and intrusive young gabbros. Note that the metavolcanics are represented mainly by metabasalts, while arc-related metasediments are classified into three types: tremolite schists, staurolite-kyanite-biotite schists, and mylonitic schists.
3. There were over 75 rock samples that were suitable for Landsat-9 sampling were collected, reflecting the various geological units in the region. The authenticity and correctness of the Landsat-9 images were demonstrated when the field samples were compared to the findings of the Landsat-9 images, the new approaches were successful in mapping the research area's rocks, as evidenced by a comparison between the findings from the suggested Landsat-9 methods and the fieldwork. This suggests that petrographic mapping of the Arabian-Nubian Shield and other dry locations may be done quickly and affordably using these approaches.
4. In this study, the spatial distribution of the basement rocks was investigated by using Landsat-9 data and the geospatial technologies of principal component analysis and band ratio (BR).
5. Based on the deep learning algorithm, AlexNet, were applied to produce an accurate, well-detailed geological map. The CNN algorithm reported an overall accuracy of 95.27% and a Kappa coefficient of 0.94.
6. The cartographic approach presented to generating results is interesting and reasonably validated by existing fieldwork and geological maps.
7. The study indicates that the use of CNN would enhance remote predictive mapping as an effective tool for regions with difficult access.
8. Future research will aim to use other types of remote sensing data, such as high spatial resolution images, to better assess the accuracy of this network architecture.

## Data Availability

Further inquiries can be directed to the corresponding author.
